# Prevalence, Antimicrobial Resistance, and Associated Factors of *Streptococcus pneumoniae* Colonization Rate among Old-Age Patients with Respiratory Tract Infection Attending Sheik Hassan Yebere Referral and Karamara General Hospitals, Jigjiga, Ethiopia

**DOI:** 10.1155/2022/9338251

**Published:** 2022-09-23

**Authors:** Surafel Mekuria, Ayichew Seyoum, Zerihun Ataro, Tigist Abebe, Kedir Urgessa

**Affiliations:** ^1^Department of Medical Laboratory Sciences, Jigjiga University, Jigjiga, Ethiopia; ^2^Department of Medical Laboratory Sciences, College of Health and Medical Sciences, Haramaya University, Harar, Ethiopia

## Abstract

**Background:**

*Streptococcus pneumoniae* is part of the normal flora of the upper respiratory tract of humans. Colonization of the upper respiratory tract (carriage of pneumococcus) by *S. pneumoniae* is considered a prerequisite for pneumococcal infection. It is the major cause of respiratory tract infection and frequent cause of physician visits, hospitalization, and death among old-aged patients because of their low immunity status. However, data on *S. pneumoniae* among old-aged patients in eastern Ethiopia are limited. This study was undertaken to determine the prevalence, antimicrobial resistance, and associated factor of *S. pneumoniae* colonization among old-aged patients.

**Method:**

A health facility-based cross-sectional study was conducted from 1 March to 15 April 2020, at Sheik Hassan Yebere Referral and Karamara General Hospitals, Jigjiga, eastern Ethiopia. A total of 188 individuals greater than or equal to 60 years suspected of both upper and lower respiratory tract infections were included. Sociodemographic, behavioral, living conditions, and clinical data were collected by trained data collectors. Sputum samples were collected and examined for *S. pneumoniae* using the culture and biochemical tests as per the standard procedures. The Kirby–Bauer disk diffusion method was used for antimicrobial susceptibility testing. The data were entered on Epi-data version 3.1, and frequencies, crude odds ratio, and adjusted odds ratio were analyzed using SPSS version 20.

**Results:**

The prevalence of *S. pneumoniae* colonization rate among old-aged patients was 13.8% (26/188) (95% CI: 9.6–19.1). Smoking (AOR = 3.3; 95% CI: 1.3–8.3), upper airway problems (AOR = 4.1; 95% CI: 1.1–15), and asthma disease (AOR = 3.1; 95% CI: 1.1–8.9) were the factors associated with *S. pneumoniae* colonization. The isolated organisms showed high antimicrobial resistance to trimethoprim-sulphamethoxazole (*n* = 12, 46.2%), tetracycline (*n* = 11, 42.3%), and ampicillin (*n* = 9, 34.6%).

**Conclusion:**

This study showed that high prevalence of *S. pneumoniae* and antimicrobial resistance for trimethoprim-sulphamethoxazole, tetracycline, and ampicillin when compared to similar studies. Cigarette smoking, having upper airway problem, and asthma disease were factors associated with *S. pneumoniae* colonization. The provision of pneumococci conjugate vaccination and avoiding smoking are highly recommended for old aged in the community.

## 1. Introduction

*S. pneumoniae* is part of the normal flora of the upper respiratory tract of humans [1]. Colonization of the upper respiratory tract (Carriage of pneumococcus) by *S. pneumoniae* is considered a prerequisite for pneumococcal disease [1, 2]. Nasopharyngeal colonization by *S. pneumoniae* is considered a prerequisite for the disease but unlike in children, colonization in the elderly is rarely detected. The incidence of pneumococcal disease is disproportionally high in infants and the elderly [3]. *S. pneumoniae* is the main bacterial cause of respiratory infections in children and the elderly worldwide [4].

*S. pneumoniae* is a Gram-positive, nonmotile, non-spore-forming, coccoid bacteria [5]. It has a cell wall containing a peptidoglycan layer and teichoic acid in larger amounts. The cell wall is covered by a polysaccharide capsule that is immunogenic and its polysaccharide composition differs among strains [6]. The bacteria on Gram stain, appear in pairs, formerly named diplococci pneumonia, but now due to its appearance in single and chain, it was *named S. pneumoniae* in 1974 [7].

Pneumococci are spread via direct contact with secretions from carriers, via saliva, or inhaled via an aerosol, where they colonize the nasopharynx [8]. Its pathogenesis is mediated by the host's response to infection by the organism. Colonization occurs when the organism gains access to the upper respiratory tract and binds to the host epithelial cells [9].

The *S. pneumoniae* bacteria are commensal to the upper respiratory tract but they can cause infection in old-aged patients due to their low immunity status [10]. Pneumococcal colonization is high, especially under conditions of crowding such as hospitals, jails, and having more family members in a single room. Generally, the associated risk factors are age, crowding, respiratory tract infection, cigarette smoking, and Siblings [11].

Old-aged peoples are prone to the infection causing an outpouring of fluid in the infected part of the lungs, due to basic disorders like an impaired function of mucociliary clearance, decreased chest wall mobility, and also reduction of blood flow to the infected portion of the lung [12].

Pneumonia is the third most common cause of hospital admission in developed countries and consumes a great proportion of the health care budget because it is a frequent cause of physician visits, hospitalization, and death among older adults [13]. The mortality rate of pneumonia caused by *S. pneumoniae* infection among old-aged patients reaches up to 40% in the world [14]. Global pneumonia death in 2017 increased among people who are 70 years and older, 1.13 million died from pneumonia were in this age group [15]. Deaths caused by *S. pneumonia* are could be easily preventable and treatable with simple and cost-effective interventions if treated early [16].

The case fatality rate reported in Australia due to *S. pneumoniae* all types of infection among age groups 65 years were 118,553 death which accounts for the crude fatality rate of 6.1% [17]. In Japanese old-aged patients in 2016, 119 650 people died due to *S. pneumoniae* and this disease was the third leading cause of death and prolonged hospitalization which lead to high treatment costs [18]. In Europe, mortality rates for pneumonia are substantially higher in old-aged patients aged 75 and over than in adult age groups, similarly, in Western Europe, the highest mortality rates for pneumonia are in elderly people aged 80 and over accounting for 279 deaths per 100 000 people [19].

In sub-Saharan Africa, pneumonia ranks among the top three diagnosed infections in hospital admissions and the fifth largest killer in South Africa, accounting for 3.9% of all deaths [20] and Similarly 10% of all death in Kenya [21] In Ethiopia, a study conducted in 2013 among the top three causes of medical impoverishment, pneumonia was the second leading cause [22]. In the other study conducted in Jimma, Ethiopia, the mortality rate of patient hospitalization due to pneumonia was 20.2%, for this age older than 65 years and other comorbid conditions among old ages aggravated poor treatment outcomes leading to death [23].

People in Jigjiga and neighboring districts are highly exposed to pneumonia disease because of the crowded living condition of the community. However, to the best of the investigators' knowledge, data are lacking on the *S. pneumoniae* colonization and antibiotic resistance in all age groups found in Jigjiga. Therefore, the aim of this study was to determine the prevalence, antimicrobial resistance, and associated factor of *S. pneumoniae* colonization among old aged patients attending Sheik Hassan Yebere Referral and Karamara General Hospitals, Jigjiga, Ethiopia. *S. pneumoniae* is a major cause of community-acquired pneumonia regarding on this the study participants came from the community so the result will be inferred for the community.

## 2. Materials and Methods

### 2.1. Study Setting, Design, and Period

A health facility-based quantitative cross-sectional study was conducted at Sheik Hassan Yebere Referral and Karamara General Hospitals from March 1 to April 15, 2020. These hospitals are giving health services to residents of Jigjiga town and patients who are referred from different districts in the region. Jigjiga town is the capital city of the Somali regional state of Ethiopia. The town is located east of 625 km away from the capital city of Ethiopia, Addis Ababa. The town is bordered in the west by Hadew, in the south by Qebribeyah, in the northeast by Awbere and in the southwest by Gursum, and southeast by Ajersagoro. The city has an elevation of 1,934 meters above sea level and it is traditionally the seat of the Bartire Garad Wiil-Waal of the Jidwaaq Absame. Religion structure in the city of Jijiga is predominantly Muslim. The ethnic composition of the town was 61.58% Somali, 23.25% Amhara, 7.32% Oromo and 4.37% Gurage, and 1.48% Tigrayan; all other ethnic groups made up 1.99% of the population [24].

### 2.2. Study Population

The source population is all patients with age ≥ 60 years old who visit the hospitals in Jigjiga. The study included all patients aged ≥60 years old who had visited Sheik Hassan Yebere Referral and Karamara General Hospitals who were suspected of both upper and lower respiratory tract infections during the study period.

### 2.3. Eligibility Criteria

Patients who were 60 years or older were included in the study. Because one of the inclusion criteria for this study was old age so old age for Africa is age ≥60 [25]. Patients were excluded if they were on antibiotic treatment, and who were critically ill and unable to undergo the assessment.

### 2.4. Sample Size

The number of study participants needed for this study was calculated based on the study from Jimma (Southwest Ethiopia) which reported a prevalence of 12.8% for *S. pneumoniae* [26]. The sample size was calculated by using the single-population proportion formula (*n* = *Z*2 *P* (1 − *P*)/*d*2) with the following parameters: the level of statistical significant set up at the level of 95% confidence interval (*Z*) (1.96) and margin of error (5%). Based on this, the minimum estimated sample size was 171. After adding 10% for nonresponse, we obtained a total of 188.

### 2.5. Sampling Procedure

Of four health institutions found in Jigjiga town, the two hospitals, namely, Sheik Hassan Yebere referral and Karamara General Hospitals were selected by purposeful sampling method. Out of the available 18 service units found in the Sheik Hassan Yebere Referral, four of them were selected by simple random sampling technique, namely, outpatient department (OPD) 2, OPD 3, Medical ward, and Emergency. Among 10 available service units in Karamara General Hospital, 4 of them (OPD 1, OPD 2, Medical Ward, and OPD 3) were selected by simple random sampling. The final calculated sample size for the study was proportionally allocated to each selected hospital's service unit. The study participants from each service unit were recruited using a convenient sampling method.

### 2.6. Data Collection

Socio-demographic data such as sex, age, occupation, educational status, number of family members, contact with a pet, behavioral characteristics such as alcohol consumption, cigarette smoking, oral hygiene, previous antibiotic use, and clinical data like malnutrition, immunization status, previous respiratory tract infection, diabetes, cardiac disease, asthma, liver disease, renal disease, tonsillectomy and HIV infection were collected by interview using a structured questionnaire and by reviewing the medical history.

### 2.7. Anthropometric Measurement

Body weight was determined to the nearest 0.1 kg on an electronic digital scale (Seca, made in Germany, quality control No: 5106, max: 150 kg, mcd 3811021659), and the participants were measured wearing light clothes and barefoot. The weighing scale was checked every morning with a 10 kg weight and calibrated to zero before taking every measurement. The standing height was obtained by measuring subjects standing on a flat surface, with their heels.

### 2.8. Specimen Collection, Handling, and Transportation

Five ml sputum specimen was collected using sterile, wide-mouth containers. The sputum sample is easily obtained from the study participant and it is representative of the lower respiratory tract infection suspected patient. Prior to collection, the specimen container was labeled with the appropriate identifying label then the cup was given to the patient after giving brief instructions on how to collect a representative sputum sample. The collected sputum specimens were placed in a leak-proof biohazard bag with sealed lids and absorbent material and transported to the laboratory in a cooler with chilled ice packs to maintain the temperature of 2–8°C.

### 2.9. Isolation and Identification of *S. pneumoniae* Bacteria

The sputum specimen was inoculated on blood agar supplemented with 5% sheep blood and the plates were incubated at 37°C with 5% CO_2_ for 18–24 hours. Colonies that showed alpha hemolysis (green zone surrounding colonies) on blood agar were further identified by biochemical tests like the fermentation of glucose, lactose, and sucrose and sensitivity for optochin disk showing ≥ 14 mm zone of inhibition. A bile solubility test was performed for those who showed ambiguous results on the Optochin susceptibility test (showing <14 mm zone of inhibition). The colonies of each representative isolate were characterized using the standard bacteriological method [27].

### 2.10. Microscopic Examination

Gram stain was performed for the suspected isolated organism from the media and it was visualized by using 100% oil objective for the suspected diplococci (pneumococci).

### 2.11. Antimicrobial Susceptibility Test

An antimicrobial susceptibility test was done on *S. pneumoniae* isolates by using the disk diffusion method on Mueller Hinton agar supplemented with 5% sheep. The zone of inhibition was measured by using a measuring ruler. The antibiotics used were erythromycin (15 *μ*g), trimethoprim-sulphamethoxazole (25 *μ*g), oxacillin (1 *μ*g), ampicillin (10 *μ*g), ceftriaxone (30 *μ*g), tetracycline (TE) (30 *μ*g), and chloraphenicol (30 *μ*g). These antibiotics were the most commonly prescribed in the study area among the standard. About 4-5 pure colonies from isolated colonies were transferred to a tube containing 5 ml sterile normal saline. The turbidity of the broth was equilibrated to match 0.5 McFarland standards. The zone of inhibition was measured by a ruler, then recorded and compared with CLSI (Clinical and Laboratory Standards Institute) technique [28].

### 2.12. Data Quality Control

The questionnaire was first prepared in English and translated into two local languages (Amharic and Somali) and then translated back to English by different bilingual experts to check the consistency. The questionnaire was pretested on 5% of the sample size in Ablelle Health Center to check the practicability and the applicability of the questionnaire. Data collectors and supervisors were trained on the objective of the study, interviewing techniques, and data quality management. The quality of each new batch of prepared culture medium and antimicrobial disks was checked by testing control strains *S. pneumoniae* ATCC (American Type Culture Collection) 49619. All testing results obtained from the reference strains were within the established quality control limits of the CLSI guideline [28].

### 2.13. Operational Definitions

  Old age: the age of a patient according to the standard of WHO whose age is greater than or equal to 60 years old [29]
  Respiratory tract infection: infection of the upper and lower respiratory tract which leads to pneumonia disease [30]
  Colonization: carriage of microorganisms

### 2.14. Data Processing and Analysis

The data were entered into Epi-data version 3.1, cleaned and analyzed using SPSS version 20. Descriptive statistics like proportion, frequency, mean, and standard deviation for an independent variable were calculated. The odds ratio was used to assess the association and statistical significance between independent and dependent variables. All variables were analyzed in the bivariate logistic regression and a variable with a *p* value ≤ 0.25 was further considered for multivariable analysis to control confiding variables. The crude odds ratio and adjusted odds ratio (AOR) was analyzed with a 95% confidence interval (CI) and a *p* value ≤ 0.05 was considered a statistically significant association with *S. pneumoniae* colonization.

### 2.15. Ethical Considerations

The study was performed as per the Helsinki Declaration and Ethiopian research regulations Ethical approval was obtained from the Institutional Health Research Ethics Review Committee (IHRERC), Health and Medical Science College, Haramaya University with Reference number IHRERC/034/2020. In addition, an official letter was obtained from Haramaya University to Karamara General and Sheik Hassan Yebere Referral Hospitals to get permission. Study subjects were given information on the study's objectives, and informed consent was obtained in writing or by thumbprint. Issues of confidentiality were maintained by keeping patient history as per medical law. Finally, study participants with positive laboratory results of *S. pneumoniae* were linked to attending physicians to get appropriate treatment.

### 2.16. Limitation

Since the study is cross-sectional it does not show cause and effect between dependent and independent variables. The study design was also susceptible to biases such as nonresponse bias and recall bias but I was trying to aware my respondents to participate in the study willingly by clarifying the study usage. There was not enough available literature on this particular topic in Ethiopia which made less for comparison locally.

## 3. Results

### 3.1. Socio-Demographic Characteristics of the Study Participants

A total of 188 old-aged patients (100% response rate) were included in the study. The majority of study participants were male 115(61.2%), Somali by ethnicity 125 (66.7%), married 141 (75%), and illiterate 111 (59%) (Table 1).

### 3.2. Behavioral and Lifestyle Characteristics

Out of the 188 participants, 41 (21.8%) and 33 (17.6%) were overweight and underweight, respectively. Fifty-one (27.1%) and 16 (8.5%) of the participants had a habit of smoking and alcohol drinking, respectively. More than half of the participants 105 (55.9%) do not brush their teeth daily, and 62 (33%) of them had contact with pets (Table 2).

### 3.3. Clinical Characteristics

Among 188 study participants, 121 (64.4%) of the study participants had upper airway problems in all cases and 36 (19.1%) of them had contact with people who had a respiratory infection. The comorbid disease commonly encountered was diabetes (30.3%) followed by renal disease (18.6%) and tonsillectomy (16.0%) (Table 3).

### 3.4. *S. pneumoniae* Colonization

The overall prevalence of *S. pneumoniae* colonization among old-aged patients attending Sheik Hassan Yebere Referral and Karamara General Hospitals was 26/188 (13.8%) (95% CI: 9.6–19.1). Bivariate and multivariable logistic regression analyses on the associated factors of *S. pneumoniae* colonization are shown in the table below (Table 4). In the multivariable regression analysis, smoking (AOR = 3.4, 95% CI 1.3–8.5), having upper airway problems (AOR = 4.3, 95% CI: 1.2–15) and asthma disease (AOR = 3.1, 95% CI 1.1–8.8) were the factors significantly associated with *S. pneumoniae* colonization.

### 3.5. Antimicrobial Susceptibility Testing

Antimicrobial susceptibility patterns were determined for all 26 isolates of *S. pneumoniae* to seven antimicrobial agents. Of all isolates, only four were susceptible to all the seven antibiotics tested. Majority of *S. pneumoniae* isolated in this study were resistant to trimethoprim-sulphamethoxazole 12 (46.2%), tetracycline 11 (42.3%), ampicillin 9 (34.6%), oxacillin 5 (19.2%), ceftriaxone 5 (19.2%), and chloraphenicol 2 (7.7%) (Table 5).

Multidrug-resistant *S. pneumoniae* is increasingly being reported from many parts of the globe [31]. Penicillin susceptibility is an important marker for the presence or absence of a multidrug-resistant phenotype. Strains with reduced susceptibility to penicillin are usually cross-resistant to other antibiotics also [32]. Prolonged colonization and rapid reacquisition provide an increased chance of exposure to antibiotics; and thus, may be an important selective factor in predisposing to antibiotic resistance [33]. In our study, 5 (19%) were resistant to only one antibiotic, 10 (38%) were resistant to two antibiotics and 6 (23%) were resistant to three or more antibiotics (Table 6).

## 4. Discussion

*S. pneumoniae* is the major cause of respiratory tract infection among old-age people due to changes in physiological and immunological status [12]. In this study, the prevalence of *S. pneumoniae* colonization among old-aged patients was 13.8% which was similar to the cross-sectional study conducted in Jimma, southwest Ethiopia (13%) [26]. However, the present prevalence was lower than in studies conducted in Netherland (22%) [2], and India (16%) [34]. On the other hand, the prevalence of the present study was higher than the study conducted in Indonesia (3%) [35], Italy (9.8%) [36], and Thailand (8%) [37]. Similarly, the study conducted in Arbaminch, South Ethiopia reported a prevalence of 11.8% for *S. pneumoniae* colonization [38], which is slightly lower than the present study. Variations in the study design, detection method, geographical difference, and implementation of vaccination status might contribute to the difference in the prevalence in different countries.

This finding showed that cigarette smoking increased the risk of *S. pneumoniae* colonization by 3.4 times. This result was higher compared with the studies conducted in Brazil [39, 40]. Generally, bacterial colonization of the lower respiratory tract is more prevalent in smokers than nonsmokers, and mucociliary clearance is defective in smokers, owing to a reduction in ciliary beat frequency, changes in volume, and viscoelastic properties of respiratory secretions [41].

Patients who had upper airway problems in the last one year were 4.3 times more likely to develop *S. pneumoniae* colonization than those who did not have. This was similar to the study conducted in Kenya [42], Tigray, Ethiopia [43], and India [44]. In this study, old-age patients who had asthma disease were 3.1 times more likely to develop *S. pneumoniae* colonization. This result was similar to studies conducted in Alberta [45], Korea [46], and Spain [47].

In this study, out of the total *S. pneumoniae* isolated from the collected samples, resistance to some commonly used antimicrobials was observed. The study revealed high resistance to trimethoprim-sulphamethoxazole (46.2%), tetracycline (42.3%), ampicillin (34.6%); and moderate resistance to oxacillin (19.2%), ceftriaxone (19.2%), and chloramphenicol (7.7%). This finding was comparable with previous studies conducted in different parts of the world. The study conducted in Korea showed comparable *S. pneumoniae* resistance to oxacillin (22.8%), and lower resistance to ceftriaxone (5.2%) compared to the present study [48] Unlike the current study, the majority of *S. pneumoniae* isolated at Felege Hiwot referral hospital, Northwest Ethiopia showed a higher level of resistance for tetracycline (45%) and oxacillin (56.7%) [49]. The other study conducted in Gonder (northwest Ethiopia) showed lower resistance to *S. pneumoniae* for ceftriaxone (9.8%) compared to the present study [50]. The difference in antimicrobial susceptibility compared to other studies might be due to geographical differences. A wide geographical variation in the antimicrobial susceptibility of *S. pneumoniae* can be explained by different bacterial strains, the availability of the drugs in clinical practice, and the misuse of antibiotics [51].

The strengths of this study include that, it provides the opportunity of assessing the prevalence of *S. pneumoniae* colonization in a rarely studied population (old-aged patients) in the context of resource-constrained countries where a study on this topic is rare. However, there are some limitations to the present study. As a result of the cross-sectional design of the study, it could not show cause and effect between dependent and independent variables.

## 5. Conclusion

The prevalence of *S. pneumoniae* colonization rate among old-aged patients was considerably high. Our finding indicated that old age patients who were smokers, and those who had asthma disease and upper airway problems were at higher risk to be infected with *Streptococcus pneumonia*. Higher *S. pneumoniae* drug resistance was observed for trimethoprim-sulphamethoxazole followed by tetracycline and ampicillin. Based on our findings, we recommend the provision of health education for the community, especially for old-age patients to take immunization against *S. pneumoniae* and to avoid risky behavior such as smoking. Furthermore, the regional health office must expand the pneumococci conjugate vaccination in the community and revise the local treatment guideline for pneumonia. The health professionals who are working in the hospitals can use erythromycin, chloraphenicol, and ceftriaxone for the treatment of infected patients with isolated bacteria.

## Figures and Tables

**Table 1:** Socio-demographic characteristics of old-aged patients attending Sheik Hassan Yebere referral and Karamara General Hospitals Jigjiga, Ethiopia, 2020 (*N* = 188).

Variables	Category	Frequency	Percentage
Age	60–64	66	35.1
	65–69	57	30.3
	70–74	27	14.4
	≥75	38	20.2

Sex	Male	115	61.2
	Female	73	38.8

Religion	Muslim	136	72.3
	Orthodox	44	23.4
	Protestant	7	3.7
	Catholic	1	0.5

Ethnicity	Somale	125	66.5
	Amhara	32	17.0
	Oromo	17	9.0
	Gurage	6	3.2
	Tigre	7	3.7
	Others	1	0.5

Educational status	Illiterate	111	59.0
	Primary	55	29.3
	Secondary	12	6.4
	College/University	10	5.3

Marital status	Married	141	75.0
	Widowed	37	19.7
	Divorced	9	4.8
	Single	1	0.5

Occupation	Farmer	45	23.9
	Merchant	43	22.8
	Housewife	39	20.7
	Government employee	12	6.4
	On pension	26	13.8
	Unemployed	23	12.2

Household members	Less than or equal to 5	52	27.7
	6–10	128	68.1
	>10	8	4.3

**Table 2:** Behavioral and lifestyle characteristics of old-aged patients attending Sheik Hassan Yebere Referral and Karamara General Hospitals Jigjiga, Ethiopia, 2020 (*N* = 188).

Variables	Category	Frequency	Percentage
BMI(Body mass index)	Underweight	33	17.6
	Overweight	41	21.8
	Normal	114	60.6

Smoking	Yes	51	27.1
	No	137	72.9

Drinking alcohol	Yes	16	8.5
	No	172	91.5

Brushing teeth daily	Yes	83	44.1
	No	105	55.9

Eating habits per day	Once	1	0.5
	Twice	16	8.5
	Three times or more	171	91

Contact with pet	Yes	62	33
	No	126	67

**Table 3:** Clinical Characteristics of old-aged patients attending Sheik Hassan Yebere Referral and Karamara General Hospitals Jigjiga, Ethiopia, 2020 (*N* = 188).

Clinical characteristics	Response	Frequency	Percentage
Upper airway problems in the last one year	Yes	121	64.4
	No	67	35.6
Contact with people who had a respiratory infection	Yes	36	19.1
	No	152	80.9
Asthma disease	Yes	27	14.4
	No	161	85.6
Renal disease	Yes	35	18.6
	No	153	81.4
Diabetes	Yes	57	30.3
	No	131	69.7
Cardiac disease	Yes	23	12.2
	No	165	87.8
Tonsillectomy	Yes	30	16.0
	No	158	84.0

**Table 4:** Bivariate and multivariable analyses for factors associated with *S. pneumoniae* colonization among old-aged patients attending Sheik Hassan Yebere Referral and Karamara General Hospitals Jigjiga, Ethiopia, 2020 (*N* = 188).

Variables	Category	*S. pneumonia*		Bivariate analysis		Multivariable analysis	
		Positive *n* (%)	Negative *n* (%)	COR(95% CI)	*P* value	AOR(95% CI)	*P* value
Educational status	Illiterate	14 (12.6%)	97 (87.4%)	0.7 (0.2–2.2)	0.49	0.7 (0.2–2.6)	0.61
	Primary	8 (14.6%)	47 (85.5%)	0.8 (0.2–2.9)	0.69	0.9 (0.2–3.5)	0.83
	Secondary and above	4 (18.2%)	18 (81.8%)	1		1	

BMI	Under weight	10 (30.3%)	23 (69.7%)	3.04 (1.1–8.2)	0.03	2.9 (0.9–8.9)	0.05
	Over weight	7 (17.1%)	34 (82.9%)	1.5 (0.5–4.4)	0.4	2.0 (0.6–6.3)	0.23
	Normal	9 (7.9%)	105 (92.1%)	1		1	

Smoking	Yes	13 (25.5%)	38 (74.5%)	3.26 (1.3–7.6)	0.01	3.4 (1.3–8.5)^*∗*^	0.01
	No	13 (9.5%)	124 (90.5%)	1		1	

Contact with pets	Yes	12 (19.4%)	50 (80.6%)	1.9 (0.8–4.45)	0.12	1.6 (0.5–5.3)	0.43
	No	14 (11.1%)	112 (88.9%)	1		1	

Upper airway problem	Yes	23 (19%)	98 (81%)	5.0 (1.4–17.0)	0.01	4.3 (1.2–15)^*∗*^	0.02
	No	3 (4.5%)	64 (95.5%)	1		1	

Asthma	Yes	8 (27.6%)	21 (72.4%)	2.9 (1.1–7.7)	0.02	3.1 (1.1–8.8)^*∗*^	0.02
	No	18 (11.3%)	141 (88.7%)	1		1	

Tonsillectomy	Yes	2 (6.7%)	28 (93.3%)	0.39 (0.08–1.7)	0.23	0.68 (0.13–3.7)	0.65
	No	24 (15.2%)	134 (84.8%)	1		1	

Abbreviations: n, number; COR, crude odds ratio; AOR, adjusted odds ratio; CI, confidence interval.^*∗*^Variables that showed significant association with *S. pneumoniae* colonization (p value < 0.05).

**Table 5:** Antimicrobial susceptibility pattern of *S. pneumoniae* isolated from old-age patients who attended Sheik Hassan Yebere Referral and Karamara General Hospitals Jigjiga, Ethiopia from March 1 to April 15, 2020 (*N* = 26).

Antimicrobial agents	*S. pneumoniae (n* = 26)		
	Resistant	Intermediate	Susceptible
	*n* (%)	*n* (%)	*n* (%)
Erythromycin	0	1 (3.1%)	25 (96.2%)
Trimethoprim-sulphamethoxazole	12 (46.2%)	0	14 (53.8%)
Ampicillin	9 (34.6%)	1 (3.8%)	16 (61.5%)
Ceftriaxone	5 (19.2%)	0	21 (80.8%)
Tetracycline	11 (42.3%)	0	15 (57.7%)
Chloraphenicol	2 (7.7%)	0	24 (92.3%)
Oxacillin	5 (19.2%)	2 (7.7%)	19 (73.1%)

**Table 6:** MDR (Multidrug Resistance) *S. pneumoniae* isolated from old age patients who attended Sheik Hassan Yebere Referral and Karamara General Hospitals Jigjiga, Ethiopia from March 1 to April 15, 2020 (*N* = 26).

	Antimicrobial agents	Resistant
		*n* (%)
Double	Ampicillin + oxacillin	2 (7.6%)
	Ampicillin + tetracycline	2 (7.6%)
	Ceftriaxone + tetracycline	1 (3.8%)
	Tetracycline + chloraphenicol	1 (3.8%)
	Ampicillin + chloraphenicol	1 (3.8%)
	Trimethoprim-sulphamethoxazole + tetracycline	2 (7.6%)
	Trimethoprim-sulphamethoxazole + ampicillin	1 (3.8%)
Multiple	Trimethoprim-sulphamethoxazole + ceftriaxone + tetracycline	2 (7.6%)
	Trimethoprim-sulphamethoxazole + Ceftriaxone + Tetracycline + oxacillin	1 (3.8%)
	Trimethoprim-sulphamethoxazole + ampicillin + tetracycline	2 (7.6%)
	Trimethoprim-sulphamethoxazole + ampicillin + tetracycline + oxacillin	1 (3.8%)

## Data Availability

The raw dataset used/analyzed during the current study is available from Mr. Surafel Mekuria upon reasonable request.

## Abbreviations

ATCC: American Type Culture Collection
BMI: Body mass index
CLSI: Clinical and laboratory standards institute
IHRERC: Institutional Health Research Ethics Review Committee
MDR: Multidrug resistance
OPD: Outpatient department.
